# Oral cancer over six decades: a multivariable analysis of a clinicopathologic retrospective study

**DOI:** 10.1590/0103-6440202305264

**Published:** 2023-12-22

**Authors:** Natália Batista Daroit, Lucas Nunes Martins, Alan Ballardin Garcia, Alex Nogueira Haas, Fábio Luiz Dal Moro Maito, Pantelis Varvaki Rados

**Affiliations:** 1 Oral Pathology - School of Dentistry, Atitus Educação, Porto Alegre, Rio Grande do Sul, Brazil; 2 School of Dentistry, Atitus Educação, Porto Alegre, Rio Grande do Sul, Brazil.; 3 Periodontology, Universidade Federal do Rio Grande do Sul, Porto Alegre, Rio Grande do Sul, Brazil; 4 Celular and Molecular Biology, Pontifícia Universidade Católica do Rio Grande do Sul, Porto Alegre, Rio Grande do Sul, Brazil.; 5 Oral Pathology - School of Dentistry, Universidade Federal do Rio Grande do Sul, Porto Alegre, Rio Grande do Sul, Brazil.

**Keywords:** mouth neoplasms, epidemiology, risk factors, squamous cell carcinoma, multivariate analysis.

## Abstract

Studies have reported changes in the epidemiological profile of patients with oral cancer in recent decades, especially regarding gender and age. This study aimed to evaluate a historical series of oral malignant lesions prevalence over six decades and define characteristics associated with the occurrence, mainly, of oral squamous cell carcinoma (OSCC). A retrospective review of histopathological records from 1953 to 2019 was conducted in three oral pathology laboratories in South Brazil about age, sex, anatomical site, clinical aspect, and histopathological diagnosis. Descriptive and analytical analyses were performed comparing the histopathological diagnoses with other variables. Multivariable logistic regression was applied to determine the associated predictors of OSCC. Of the 53,065 records available in the institutions, 986 were oral malignant tumors (including all malignant neoplasms), representing 1.86% of all diagnoses. The occurrence of OSCC in the 1960's was 80.0%, decreasing over time reaching the lowest percentage of cases in the 1990's (75.8%) and significantly increasing to 88.7% in the 2010s. Females had a lower chance than males of having OSCC independently of the decade (odds ratio=0.30, p<0.001). This was the same for older individuals compared to those younger than 40 years. No interactions between sex, age, and decade were observed. The number of diagnoses of oral malignant lesions increased over time, and the occurrence of OSCC varied. Older individuals and males had higher chances of having OSCC independently of the decade. Therefore, in this study sample, no changes were observed in the epidemiological profile over the years concerning these predictors.

## Resumo

Alguns estudos relataram mudanças no perfil epidemiológico dos pacientes com câncer bucal nas últimas décadas, principalmente quanto ao gênero e a idade. Este estudo teve como objetivo avaliar, em uma série histórica, a prevalência de todas as lesões malignas bucais ao longo de seis décadas e definir características associadas na ocorrência, principalmente, de carcinoma espinocelular (CEC). Um levantamento retrospectivo dos registros histopatológicos de 1953 a 2019 foi realizado em três laboratórios de Patologia Bucal no Sul do Brasil em relação à idade, gênero, sítio anatômico, aspecto clínico e diagnóstico histopatológico. Análises descritivas e analíticas foram realizadas comparando-se os diagnósticos histopatológicos com as outras variáveis. A regressão logística multivariada foi aplicada para determinar os possíveis preditores associados ao CEC. Dos 53.065 prontuários disponíveis nas instituições, 986 eram tumores malignos bucais (incluindo todas as neoplasias malignas), representando 1,86% de todos os diagnósticos. A ocorrência de CEC na década de 1960 foi de 80,0%, diminuindo ao longo do tempo, atingindo o menor percentual de casos na década de 1990 (75,8%) e aumentando significativamente para 88,7% na década de 2010. As mulheres tiveram menor risco de desenvolver CEC do que os homens, independentemente da década (OR=0,30, p<0,001). Este foi o mesmo para indivíduos com idade mais avançada em comparação com aqueles com menos de 40 anos de idade. Não foram observadas interações entre gênero, idade e década. O número de diagnósticos de lesões malignas bucais aumentou ao longo das décadas e a ocorrência de CEC variou. Indivíduos com mais de 40 anos e do sexo masculino tiveram maiores chances de ter CEC, independentemente da década analisada. Portanto, nessa amostra estudada, não foram observadas mudanças no perfil epidemiológico ao longo dos anos com relação a esses preditores.

## Introduction

Oral cancer has been a public health problem for centuries. The estimated number of new lip and oral cavity cancer cases was 377,713 with 177,757 deaths worldwide ^1^. Different histological subtypes of malignant neoplasms of the oral cavity have been identified. Most cases are epithelial tumors, especially oral squamous cell carcinoma (OSCC), followed by others, such as salivary glands, sarcomas, odontogenic, and hematolymphoid ^2^.

Findings from the International Head and Neck Cancer Epidemiology (INHANCE) demonstrate well-established risk factors for head and neck cancer, such as alcohol and tobacco. Diet, genetic variants, oral health, low educational attainment, low income, and human papillomavirus (HPV) infection remain controversial in the literature as initiating/promoting factors for oral cancer ^3^.

Some studies have shown changes in the epidemiological profile of oral cancer. Traditionally, OSCC has been diagnosed in elderly men. However, in recent decades, an increasing incidence of oral cancer among young people has been reported ^4^,^5^. Likewise, reports have shown an increase in the incidence in women ^6^,^7^. Nevertheless, the epidemiological distribution of oral cancer about individuals’ characteristics, such as sex and age, lesion site, clinical aspect, and histopathological diagnosis may vary according to locoregional variations and cultural habits ^8^.

Over time, it is important to review the most affected groups and the most frequent lesions to define strategies for preventing, early detection, and diagnosing oral malignant lesions. In addition, retrospective studies with a long observation period aim to trace the trend of the historical series to define the profile of distribution and design future public health strategies ^9^. Therefore, this study aimed to investigate the occurrence and clinicopathologic trends of oral cancer, from 1953 to 2019, in cases diagnosed in a South Brazilian population. Additionally, multivariable models were applied to adjust the changes in the occurrence of OSCC and to determine factors associated with it.

## Materials and methods

The present analysis was based on retrospective data from three oral pathology laboratories: a public university (Universidade Federal do Rio Grande do Sul - UFRGS, 1953-2019), a private university (Pontifícia Universidade Católica do Rio Grande do Sul - PUCRS, 1972-2019) and a private institution of Oral Diagnosis (Oral Diagnosis Center - CDB, 1987 and 2019). All these institutions are located in Porto Alegre, the capital city of Rio Grande do Sul state, in south Brazil. This study was approved by the ethical committees of Atitus Educação (protocol number 22860719.5.0000.5319), PUCRS (protocol number 22860719.5.3003.5336), and UFRGS (protocol number 22860719.5.3001.5327). This study was conducted in compliance with the principles of the Declaration of Helsinki. Pathology specimens in these laboratories were obtained from procedures performed by academics within faculties supervised by professors, and general and specialist dentists of private as well as public dental practices.

Histopathological records were reviewed for the following variables: year of diagnosis, age, sex, skin color, habits (tobacco and alcohol consumption), anatomical site, clinical aspect, and histopathological diagnosis. The inclusion criteria were histopathological reports with a diagnosis of primary malignant neoplasm of the oral cavity, patients of any age, and both sexes. Diagnoses of the oral cavity and lips other than malignant neoplasms, and malignant tumors from an anatomical site other than the oral cavity and lips, were excluded from the sample.

Data were analyzed using SPSS Statistics for Windows (version 20.0. Chicago: SPSS Inc). Descriptive statistics were derived for the whole sample. Skin color, tobacco, and alcohol use could not be analyzed due to a large number of missing data. Therefore, bivariate comparisons were conducted using the Chi-square test.

The occurrence of OSCC was analyzed separately from other lesions considering sex, age, institution diagnosis, and decades variables by applying multivariable logistic regression. Odds ratios (OR) and 95% confidence intervals (95%CI) were reported. The individual was the analytical unit. Statistical significance was set at 5%.

## Results

A total of 53,065 histopathological diagnoses were reviewed over 66 years (1953-2019) at the three laboratories. Of these, 986 were malignant tumors, representing 1.86% of the sample. The mean age of the sample was 58.31 years (standard deviation 13.91 years; range 7-102). The sample consisted of 71.4% of men and 27.2% of women. Regarding tobacco and alcohol habits, more than 70% of the records did not contain this information. Regarding skin color, 200 (20.3%) reports did not contain this information, whereas 682 (69.2%) were white, and 104 (10.5%) were nonwhite.

Seven pediatric patients (< 18 years of age) with malignant neoplasms were identified, of which five were women, two were men, and all were white. The most common histopathological diagnoses in this sample were sarcomas (two fibrosarcomas, one Ewing’s sarcoma, and one fusiform cell sarcoma), followed by epithelial tumors (one ameloblastic carcinoma, one mucoepidermoid carcinoma and one carcinoma in situ). The most frequent sites of these lesions were the maxilla and mandible.

Considering the entire sample, the most affected anatomical site was the tongue with 257 (26%) of the cases, followed by the floor of the mouth with 153 (15.5%) and the mandible with 143 (14.5%). In terms of clinical aspects, most lesions were ulcers in 484 cases (49%), followed by exophytic lesions in 151 cases (15.3%) and leukoplakia in 102 cases (10.3%). A total of 795 cases of squamous cell carcinoma (80.5%) were identified in the sample. Other diagnoses more frequent were sarcomas with 29 cases (2.9%), verrucous carcinoma with 29 (2.9%), mucoepidermoid carcinoma with 25 (2.5%), and adenoid cystic carcinoma with 23 (2.3%). The descriptive analysis is shown in Table 1.

There was a significant association (p < 0.05) between the histopathological diagnoses and tumor site (Table 2). OSCC was correlated with lip, tongue, floor of the mouth, palate, mandible, and maxilla; sarcomas correlated with jaws; salivary gland neoplasms with palate, maxilla, and mandible. There was also a statistically significant, but weak (Spearman r=0.34, p<0.001), association between histopathological diagnoses and the clinical aspect of the lesion. Ulcer, leukoplakia, erythroplakia, and leukoerythroplakia were the clinical manifestations of OSCC, this diagnosis had no cystic lesion and this was statistically significant. Exophytic lesions were correlated with undifferentiated carcinoma, adenoid cystic carcinoma, acinar cell carcinoma, verrucous carcinoma, mucoepidermoid carcinoma, and OSCC. There was a significant association (p < 0.05) between the histopathological diagnoses and sex (Table 3). 

**Table 1:** Demographic and clinicopathologic distribution of malignant tumors in South Brazilian patients

Variables	Distribution
Sex	
Male	704 (71.4%)
Female	268 (27.2%)
Not Informed	14 (1.4%)
Skin Color	
White	682 (69.2%)
Non-white	104 (10.5%)
Not Informed	200 (20.3%)
Tobacco	
Yes	220 (22.3%)
No	66 (6.7%)
Not Informed	700 (71%)
Alcohol	
Yes	146 (14.8%)
No	86 (8.7%)
Not Informed	754 (76.5%)
Anatomical Site	
Tongue	257 (26%)
Floor of mouth	153 (15.5%)
Mandible	143 (14.5%)
Palate	120 (12.2%)
Lip	103 (10.4%)
Buccal mucosa	61 (6.2%)
Alveolar Ridge	58 (5.9%)
Maxilla	51 (5.2%)
Tonsillar Pillar	2 (0.2%)
Not Informed	38 (3.9%)
Clinical Aspect	
Ulcer	484 (49%)
Exophytic lesion	151 (15.3%)
Leukoplakia	102 (10.3%)
Erythroplakia	33 (3.3%)
Leukoerythroplakia	23 (2.3%)
Intraosseous	10 (1%)
Necrosis	5 (0.5%)
Cystic	2 (0.2%)
Fistula	2 (0.2%)
Not Informed	174 (17.7%)
Histopathological Diagnosis	
Epithelial	
Squamous Cell Carcinoma	795 (80.5%)
Verrucous carcinoma	29 (2.9%)
Undifferentiated Carcinoma	13 (1.3%)
Basal cell carcinoma	10 (1.0%)
Melanoma	2 (0.2%)
Cuniculatum Carcinoma	2 (0.2%)
Ameloblastic Carcinoma	1 (0.1%)
Spindle cell carcinoma	1 (0.1%)
“In Situ” Carcinoma	1 (0.1%)
Salivary Gland	
Mucoepidermoid Carcinoma	25 (2.5%)
Adenoid Cystic Carcinoma	23 (2.3%)
Adenocarcinoma N.O.S.	15 (1.5%)
Acinar cell carcinoma	12 (1.2%)
Others	
Sarcomas	29 (2.9%)
Undifferentiated Malignant Neoplasm	15 (1.5%)
Malignant Lymphoblastic Neoplasm	13 (1.3%)

**Table 2:** Distribution of the histopathological diagnoses and the tumor site.

	Lip	Tongue	Floor of the Mouth	Palate	Mandible	Alveolar Ridge	Buccal Mucosa	Maxilla	Tonsillar Pillar	Total
Squamous Cell Carcinoma	91*	244*	142*	80*	98*	42	47	22*	2	795
Verrucous Carcinoma	4	6	2	3	2	2	5*	4*	0	29
Undifferentiated Carcinoma	0	0*	3	2	4	1	1	2	0	13
Basal cell Carcinoma	3*	1	0	1	0	0	1	0	0	10
Melanoma	1	0	0	0	0	0	1	0	0	2
Cuniculatum Carcinoma	0	2*	0	0	0	0	0	0	0	2
Ameloblastic Carcinoma	0	0	0	0	1	0	0	0	0	1
Spindle cell carcinoma	0	0	0	0	0	1*	0	0	0	1
“In situ” Carcinoma	0	0	0	1*	0	0	0	0	0	1
Mucoepidermoid Carcinoma	0	0	1	11*	8*	0	2	2	0	25
Adenoid Cystic Carcinoma	1	2	1	4	3	3	2	7*	0	23
Adenocarcinoma N.O.S.	1	1	1	5*	2	0	1	2	0	15
Acinar cell carcinoma	0	0	2	0	1	1	1	0	0	5
Sarcoma	0	0	1	4	14*	2	0	6*	0	29
Undifferentiated Malignant Neoplasm	0	0	0	3	5	4	0	2	0	15
Malignant Lymphoblastic Neoplasm	0	0*	0	3	3	2	0	4*	0	13

*Significant association (p<0,05)

**Table 3:** Distribution of the histopathological diagnoses and sex

	Male	Female	Total
Squamous Cell Carcinoma	609 (86.5%) *	179 (66.8%)*	795 (81.8%)
Verrucous Carcinoma	18 (2.5%)	10 (3.7%)	29 (3%)
Undifferentiated Carcinoma	7 (1%)	5 (1.9%)	13 (1.3%)
Basal cell Carcinoma	7 (1%)	3 (1.1%)	10 (1%)
Melanoma	1 (0.14%)	1 (0.37%)	2 (0.2%)
Cuniculatum Carcinoma	1 (0.14%)	1 (0.37%)	2 (0.2%)
Ameloblastic Carcinoma	1 (0.14%)	0	1 (0.1%)
Spindle cell carcinoma	0	1 (0.37%)	1 (0.1%)
“In situ” Carcinoma	0	1 (0.37%)	1 (0.1%)
Mucoepidermoid Carcinoma	11(1.5%)*	13 (4.9%)*	25 (2.5%)
Adenoid Cystic Carcinoma	7 (1%)*	14 (5.2%)*	23 (2.3%)
Adenocarcinoma N.O.S.	8 (1.1%)	7 (2.6%)	15 (1.5%)
Acinar cell carcinoma	3 (0.42%)	2 (0.74%)	5 (0.5%)
Sarcoma	11 (1.5%)*	17 (6.3%)*	29 (3%)
Undifferentiated Malignant Neoplasm	8 (1.13%)	7 (2.6%)	15 (1.5%)
Malignant Lymphoblastic Neoplasm	7 (1%)	5 (1.9%)	13 (1.3%)
Total	704 (100%)	268 (100%)	972 (100%)

*Significant association (p<0,05)

**Figure 1:**
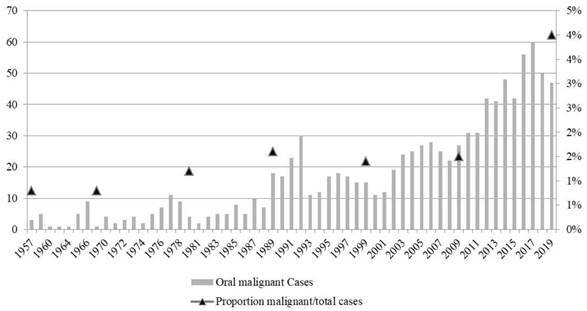
Distribution of number of cases over the years and proportion of malignant cases/total cases (%).

**Figure 2:**
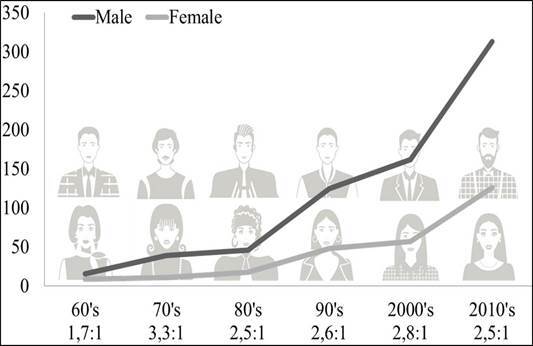
Distribution of patients across the different decades according to sex and male:female ratio.

An increase in the number of cases was observed over time. The year 2017 had the highest number of diagnoses with 60 cases (6.1%) of oral malignant lesions (Figure 1). An increase in the number of cases in men and women in recent decades was observed, and this increase was proportional, as the male-to-female ratio showed similar (Figure 2).

**Figure 3:**
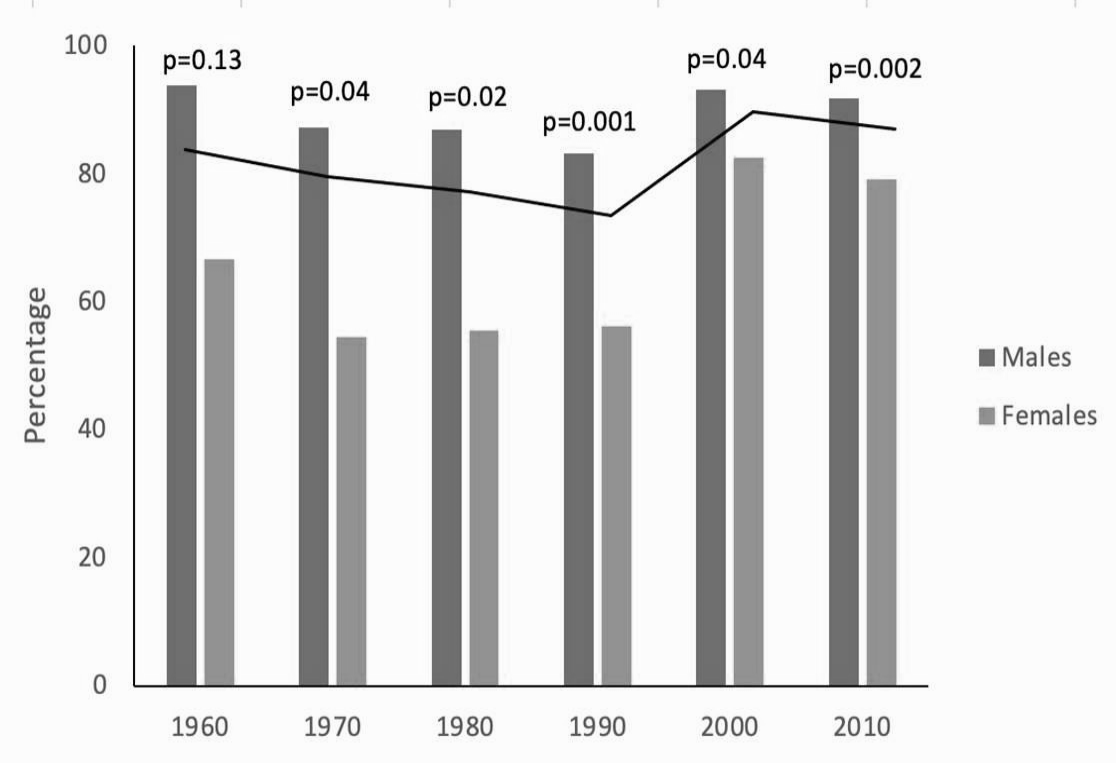
Percentage of individuals with oral squamous cell carcinoma over decades according to sex (black line indicates total percentage).

Figure 3 demonstrates the overall percentage of individuals with OSCC over the decades (black line) and according to sex. OSCC was observed in 84.6% of individuals in the 1960s, decreasing to 74.9% in the 1990s, rising to 90.5% and 87.8% in the 2000s and 2010s, respectively. These changes were statistically significant (p<0.001). Regarding the differences between sexes, there was no significant difference in 1960 (p=0.13); however, during all other decades, the occurrence of OSCC was significantly higher among males than females, and this difference did not change significantly over time.

Table 4 shows the distribution of individuals with OSCC according to sex, age, institution, and decade and the logistic regression models. Males (90.7%) had a higher occurrence of the disease than females (72.1%). After 40 years of age, the occurrence was higher than under 40 years. A higher frequency of diagnosis was observed in public than private institutions. The occurrence of OSCC in the 1960's was 80.0%, decreasing over time reaching the lowest percentage of cases in the 1990s (75.8%) and increasing after that. In the final multivariable model, females had a 70% lower chance of having OSCC than males. The chance of having OSCC increases with age. The chance of OSCC diagnosis was 59% higher in public institutions. The chances of OSCC diagnosis significantly increased after the 1990s almost threefold.

**Table 4:** Distribution of individuals with OSCC according to predictors and logistic regression models reporting odds ratio (OR) and 95% confidence interval (95%CI).

			Univariable models		Multivariable full final model	
Variables	% (SE)	p	OR	95%CI	OR	95%CI
Sex						
Male	90.7 (1.1)	Ref	1		1	
Female	72.1 (2.9)	<0.001	0.26	0.18 - 0.39	0.30*	0.20 - 0.45
Age category						
<40 years	49.3 (6.0)	Ref.	1		1	
40-49 years	88.7 (2.7)	<0.001	8.09	3.96 - 16.55	6.45*	3.03 - 13.71
50-59 years	92.3 (1.7)	<0.001	12.35	6.40 - 23.81	9.30*	4.62 - 18.74
60 years and older	86.4 (1.6)	<0.001	6.55	3.80 - 11.30	5.77*	3.20 - 10.41
Institution						
Private	80.4 (2.4)	Ref	1		1	
Public	87.9 (1.3)	0.01	1.75	1.20 - 2.57	1.59**	1.03 - 2.46
Decades						
1960	80.0 (9.1)	0.66	1.28	0.40 - 4.05	1.92	0.50 - 7.40
1970	82.5 (6.0)	0.33	1.51	0.62 - 3.67	1.77	0.65 - 4.77
1980	77.2 (5.6)	0.83	1.08	0.53 - 2.21	1.15	0.51 - 2.59
1990	75.8 (3.3)	Ref.	1		1	
2000	90.3 (2.1)	0.003	2.98	1.66 - 5.36	2.64**	1.41 - 4.94
2010	88.7 (1.5)	0.001	2.53	1.58 - 4.05	2.73**	1.63 - 4.58

SE: standard error; Ref.: reference category; *p<0.001; **p<0.05

## Discussion

Several studies have reported changes in the epidemiological profile of patients affected by oral cancer in recent decades, especially about age and sex ^4^ ^,^ ^5^ ^,^ ^6^ ^,(^ ^7^. The present study retrospectively reviewed the histopathological records of oral cancer diagnosed at three South Brazilian Oral Pathology Laboratories for more than 60 years. The findings indicated that the trend related to the greater involvement of young patients and women in recent years was not observed in this sample. There was an increase in the number of cases in these groups; however, this followed a general increase in the entire sample. Regarding OSCC cases, the risk group classical profile: males over 40 years of age, remained predominant in all the decades studied.

Analyzing the historical series, there was a proportional increase in cases of women about the number of cases of men, so the male-to-female ratio did not have a large variation. Over the last four decades, the mean rate was 2,4:1. Comparing this ratio with another study in Brazil, a smaller number was observed; the ratio observed by Moro et al ^10^ was 6,7:1. In countries such as India and Thailand, this rate is reversed, with more women affected than men with proportions of 1:2 and 1:1,5, respectively ^11^,^12^. In a multicenter intercontinental study, the mean rate was 2,2:1 ^8^.

The suggestive increase in the number of cases of OSCC in women in recent decades has been attributed to their greater exposure to risk factors (alcohol and tobacco); however, in a recent study by Wolfer et al. ^13^, most cases of women affected by OSCC were non-smokers and non-drinkers. A limitation of our study was the absence of data regarding these risk habits and, therefore, the lack of parameters to compare the historical series about these variables.

This study confirmed that OSCC predominates in men (86.5% of the sample). In this study, there was statistical significance with a female predominance for diagnosing sarcomas and salivary gland tumors, such as mucoepidermoid carcinoma (MEC) and adenoid cystic carcinoma (ACC). Gontarz et al. ^14^ observed more cases of malignant salivary gland tumors in women, especially of the histological type ACC. Wang et al.^15^ also found a female predominance for these lesions with an M:F ratio of 0.63 for MEC and 0.6 for ACC. About sarcomas, the result was contrary to that found by de Carvalho et al. (16), in which the predominance of this histological type was in general in men; however, when the subtypes were analyzed separately, the majority were found in women concerning osteosarcomas, chondrosarcomas, fibrosarcomas, and Ewing's sarcomas, and this epidemiological variation may occur due to the rarity of these lesions in the oral cavity.

The mean age was 58.31 years in all cases. However, when only OSCC cases were analyzed, the mean was 58.13 in men and 63.57 in women. Over the study period, the mean age of OSCC patients did not undergo significant variations, whereas the other diagnostics (non-OSCC) had lower means and greater variability. The same was observed by Alotaibi ^17^ in which 77.9% of non-OSCC cases were aged < 50 years old, while only 31.5% of OSCC cases were aged less than 50 years.

In the present study, 1.86 % of the samples were oral malignant tumors. Compared to other investigations, the intercontinental assessment was 1.30% (the prevalence of oral cancer ranged from 0.83% in Taiwan, 1.71% in Canada, 3.43% in Korea, 3.88% in Iran, and 6.23% in Thailand) (8). In the present study, 80.5% of patients were diagnosed with OSCC, compared with other studies Dhanuthai et al. ^8^ found 80.05%, and Alotaibi 58.3% ^17^. This variability in results may be due to regional variability in terms of risk factors.

In this study, the preferred sites for oral cancer were the tongue, floor of the mouth, mandible, palate, and lips. Epidemiological surveys have shown that the tongue has the highest prevalence of OSCC cases ^8^,^18^. In the OSCC samples, this site was observed to be the most prevalent, followed by the floor of the mouth, mandible, palate, and lip. However, there is a difference between the most prevalent sites after the tongue between Asian and non-Asian countries. Due to the habit of chewed forms of tobacco, Asian patients have greater involvement in the labial/buccal mucosa, followed by the gingiva and palate. In non-Asians, the most frequent sites were the alveolar mucosa, followed by the floor of the mouth and lip, which was similar to this sample ^8^. An interesting result of this study was the high prevalence of lesions in the mandible, in addition to a statistically significant relationship between squamous cell carcinoma and mucoepidermoid carcinoma occurring in this site. Methodologically, alveolar mucosa and gingiva were included in the "mandible" category. These data were extracted from anatomopathological examination forms, which in some cases did not specify the exact anatomic location of tumor involvement. Malignant lesions in the mandible are uncommon^8^, but considering the gingival and alveolar mucosa subsites, some studies show a greater involvement ^12^,^17^,^19^, mainly in Asian countries where the betel quid/ tobacco chewing is practiced, this habit is not observed in the studied population and could not justify this prevalence.

Regarding non-OSCC malignant tumors, a relationship between the subsites and the histopathological diagnosis was observed. In the present study, sarcomas significantly affected the jaws (mandible and maxilla), similar to the findings of de Carvalho et al. ^16^, who found the mandible, palate, and maxilla. Glandular malignant tumors, such as ACC, MEC, and adenocarcinoma NOS showed a statistically significant relationship with the palate, maxilla, and mandible in this sample. The palate is the most common location of malignant minor salivary gland neoplasms; the buccal mucosa, retromolar trigone, and lower lip are other frequent locations of these neoplasms ^20^.

Approximately half of the studied sample presented with ulcers; without a doubt, this clinical aspect should be considered a warning sign when a professional observes this type of lesion. However, considering the statistical significance between the diagnoses of OSCC, MEC, ACC, verrucous carcinoma, and undifferentiated carcinoma with exophytic lesions, malignant lesions should also be among the differential diagnoses of these lesions. Analyzing the surface of exophytic lesions, Allon et al. ^21^ observed that ulcerated masses representing malignancy were more numerous than non-ulcerated masses; however, the majority of malignancies presented as non-ulcerated masses, showing that the loss of epithelial covering should not be considered an indicator of malignancy.

There has been a significant increase in the number of oral cancer diagnoses in the samples studied over the last two decades. Comparing estimates from the National Cancer Institute (INCA) for the state of Rio Grande do Sul from 2005 (810), 2006 (800), 2008 (820), 2010 (800), 2012 (820), 2014 (1030), 2016 (1110), 2018 (1100 cases) ^22^, there was an increase of 35% between the first and last-mentioned year. In the present study, an increase of 74% was observed during the same period. This result can be explained as follows. 1) Since 2004, when the National Oral Health Policy was implemented in Brazil, compose this program Specialized Dental Care Centers (CEOs) and two establishments in oral diagnosis were opened in our region, and as a result, clinical detection and oral biopsies increased. 2) Regional professionals, dental and teaching entities, besides the government, have been promoting for over 20 years awareness campaigns; prevention, and early diagnosis of Oral Cancer entitled "Maio Vermelho.” 3) Continuing education programs for primary oral healthcare dentists increased self-efficacy for oral mucosal lesion management and encouraged these professionals to manage these cases correctly ^23^. 4) Oral pathology centers have started their activities in different periods; therefore, there has been an accumulation of cases in recent years.

Due to the lack of information in the histopathological records of oral cancer risk factors (tobacco and alcohol), it was not possible to infer from the historical series the pattern of these habits over the years. Another study performed in the same sample, but with a shorter time (1995-2004) comparing the completeness of oral biopsy forms, showed that the most frequently completed biopsy forms were associated with malignant epithelial tumors (83.2%) ^24^. In our practice, we observed that the most recent records were complete, with details of the patient’s habits, as well as complementary information on the clinical aspect of the lesions. This improvement can be due to the constant promotion of dentists' and dental students' role in studying of oral mucosal diseases and practice ^25^.

Classically, initiating and promoting oral cancer risk factors are well established, such as alcohol and tobacco in its various forms, smoke, and smokeless tobacco. Other factors have been widely studied, such as the carcinogenic potential of the human papillomavirus (HPV). Currently, it is considered a possible etiology for oropharyngeal squamous cell carcinoma, as a consensus in the literature, but not for all oral tumors. Also, genetic alterations in the genome have been reported as somatic mutations in several genes, including NOTCH1, KMT2D, CASP8, AJUBA, NSD1, HLA-A, and TGFBR2, in addition to systemic diseases having an increased risk for developing oral cancers such as Xeroderma Pigmentosum, Fanconi anemia, Congenital Dyskeratosis ^26^,^27^,^28^. The investigation of these possible risk factors involved is important in studies of different approach levels and designs to establish cause-effect relationships.

Our sample contained three oral pathology laboratory samples, one within a public university (where the patient did not have to pay for undergoing an anatomopathological examination) and the other two private institutions (where the patient had to pay for the examination). Interestingly, a higher frequency of cancer was observed in public than private institutions and the chance of OSCC diagnosis was 59% higher in public institutions. This result can be attributed to 1) a higher frequency due to the different range of time in each laboratory. 2) the co-occurrence of multiple risk behaviors (unhealthy diet, alcohol abuse, and smoking) associated with lower socioeconomic status, directly influencing individuals' education, prevention, and health promotion (29). These variables should be considered in future studies to highlight other etiologic factors in OSCC development as this information was missed in the majority of biopsy records evaluated in this research. 

The present study confirmed the epidemiological profile of oral malignant tumors throughout the historical series. White men, over 40 years of age remain the profile most affected by OSCC even in recent decades. The number of diagnoses of oral malignant lesions increased over time. These data reinforce that prevention campaigns about this disease and early detection strategies should be encouraged once patients at risk for oral cancer, especially squamous cell carcinoma, are very well defined.
